# Teaching of the Black Population’s Health: Anti-Racist Lenses for a Paradigm Shift to Address Racial Inequities

**DOI:** 10.3390/ijerph192416784

**Published:** 2022-12-14

**Authors:** Ana Paula Borges Carrijo, Anna Luísa Dias Bastos de Moura, Augusto Cézar Polveiro e Oliveira, Lígia Villela Rodrigues, Janaina de Oliveira, Thiago Figueiredo de Castro, Odete Messa Torres, Katia Crestine Poças, Rodolfo Deusdará

**Affiliations:** Faculdade de Medicina, Universidade de Brasília, UnB, Campus Universitário Darcy Ribeiro, Asa Norte, Brasilia 70910-900, DF, Brazil

**Keywords:** racism, higher education, race/ethnicity, medical curriculum

## Abstract

Health (and its dialectical pair—illness) is determined by multiple factors: social class, educational background, income, occupation, and race/skin color. Racism can directly impact physical and psychological illnesses, with an effect on social conditions of health. This paper discusses: (1) racism as a root cause of health inequities in Brazil and elsewhere, and (2) how students at the University of Brasilia School of Medicine respond to an anti-racist curriculum. We emphasize that an environment of profound exchanges in the teaching–learning process, adopting anti-racism praxis as a competency in the medical curriculum, is a paradigm shift in medical education and future practice.

## 1. Introduction

Health (and its dialectical pair—illness) is determined by multiple factors: social class, educational background, income, occupation, and race/skin color [1,2]. Contemporary medicine has been forced to overcome the biomedical paradigm to incorporate social theories and a socioeconomic approach to health problems and, in this sense, to recognize health as a human right—with emphasis on equity—ensuring care and facing inequities [1,2]. Confronting racism is a health-promoting action: a necessary and urgent public health policy [3,4].

Racism can directly impact physical and psychological illness [3,5], with an effect on social conditions of health [6]. Living conditions force the black population to a workload that limits their self-care, nutrition, and physical exercise, in addition to making it difficult to maintain support networks [6]. On top of intersubjective violence and poverty, race establishes itself as a racial determinant of health [6], which the physician must look at and care about. Structural racism—resulting from the functioning of institutions that confer disadvantages and privileges based on race [7]—produces immeasurable losses and inequities [5,6,7].

Even after 134 years of the abolition of slavery in Brazil, slavery had and still has fundamental implications for social inequalities. The black population tends to be born and raised in families with less economic and cultural resources. They face disadvantages that impact school and occupational results, and wage conditions [8]. Black people have an average household income twice as low as white people. Almost 80% of Brazilians living below the poverty line are black or brown people [9]. In 2019, almost 80% of the homicide victims were black or brown people in Brazil [10]. Although affirmative action policy led to more black people in Brazilian universities, the majority of students were white people, particularly in schools of medicine [11]. 

In light of those principles and of this reality, this work comments on the incorporation of an anti-racist praxis [3] in medical education [12] in the University of Brasilia. The purpose is to discuss possible actions to combat racism as a root cause of health inequities in Brazil and in the world [4]. 

## 2. Materials and Methods

This is a descriptive and qualitative study designed as a narrative report of an undergraduate teaching experience on Black People Health Policies at the Medical School of the Brasilia University. The path taken in one discipline of the reviewed curriculum of the Medical School [13] is contextualized with the challenges faced by this specific population in Brazil and then discussed, including the general practitioner professors’ and students’ point of view.

The discipline applies an anti-racist focus to medical teaching, bringing contents such as neglected illnesses (tuberculosis, Hansen’s disease, and arboviruses) and health of deprived populations (black, indigenous, imprisoned, street, and slum populations). The discipline was created in 2018 and is offered every semester. Each semester, approximately fifty new medical students are enrolled in the discipline, which is mandatory in the medical course. Three professors of Family Medicine are responsible for teaching the health of the black population in this discipline for fifteen weeks, every Wednesday morning for four hours, in the 3rd year of the medical course. 

When teaching about the health of the black population, professors give lectures and provide content in various formats, such as scientific articles, reference texts on national policies, books, podcasts by jurists and leaders of the black movement, among other audiovisual resources. 

From the presentation and debate of the content, the students carry out critical reviews in which they evaluate the learning experience, as well as the inclusion of new content in the teaching–learning process.

The last course week is used for students and professors to provide and receive feedback interactively. The experience of training participants is captured by interactions during the class and in the final exam. The impressions are contrasted with the challenges of struggling against structural racism in Brazil, especially in Brazilian medical education.

## 3. Challenges in Brazil

In Brazil, the Black and the Health Reform movements fought for universal social rights for the black population and equitable access to health. They achieved the creation of the Unified Health System [4,14]. However, unequal access to goods and services, racial inequality, and racism kept promoting illness [4]. 

In the case of the black population, special conditions of vulnerability are determined by the environment, such as disqualified and devalued social inclusion (social vulnerability), and by the invisibility of their real needs in assistance, health promotion, and disease prevention actions and programs (programmatic vulnerability). This way, black women and men live in a constant defensive state, which collaborates with inappropriate behavior, and psychic, psychosocial, and physical illnesses (individual vulnerability) [15].

In this country, black skin color is associated with lower levels of education and income, occupational activities with higher risks, and disparity in the access of and difficulties in health care [4,5,14]. This reality reveals that racism is related with adverse health outcomes [5] involving sociocultural and emotional factors, through stigma, stereotypes, prejudice, and racial discrimination. All of them contribute to unequal access to economic resources and various other resources and social opportunities. Moreover, racism constitutes a cause that can exacerbate the negative impacts of other risk factors for health, as it drives and sustains stressors such as discrimination and historical trauma, as well as affects other levels of stressors such as unemployment, financial difficulties, violence from neighbors, or exposure to physical and chemical agents at home and in work environments [16]. 

Despite institutional pressure for public policies aimed at confronting racism, it took decades for authorities to materialize health and educational initiatives focused on race/color [4,14]. Racial democracy in Brazil is—still—a myth, as equal rights were not fully guaranteed to black people [17]. In addition, structural racism affects institutions’ and organizations’ routines, which lead to inequalities in access to services, benefits, and opportunities to different segments of the population [18]. In the Brazilian case, the influence exerted by the idea that we are a “racial democracy” is visible [19]. In the imagination of Brazilian society, everyone has possibilities of ascension, without distinction, within the standards established by a bourgeois mindset. This “racial democracy” disregards the historical, cultural, ideological, and subjective constructions of the social, economic, and political relations of the black population, thus favoring the ruling classes, preventing or making difficult the establishment or expansion of true democracy [20].

## 4. Challenges in the Brazilian Medical Education

Medical education centered on the traditional biomedical model removes the psychic and social dimensions of education, limiting the future professional’s tools for care [21]. Teaching must provide students with an empathetic contact with the patient, making them understand the effects of racism on the subject and on the black population’s health. This movement can be done through promoting critical reading, writing, researching, and thinking of the supervised clinical care, as well as valuing students’ skills and attitudes, and building spaces that encourage discussion about an anti-racist praxis [12].

The National Curriculum Guidelines for the Medical Course (2014) incorporate the humanistic view of the patient into the technical–scientific aspects, contributing to the transition from an anatomo-clinical and hospital-centered model to the paradigm of continuous and comprehensive care for the individual [22]. They establish the need to include the sociocultural, humanistic, and biological aspects of the human being from an interprofessional and multidisciplinary approach during the medical course. 

The changes in the National Curriculum emphasize that general practitioners training in primary care and urgency/emergency settings should meet the health needs of the population. For this purpose, the Guidelines are centered on three major areas: Integral Health Care, Health Education and Health Management. These areas are intended to contribute to the development of skills that allow for greater aptitude in dealing with the problems of Brazilian society and public health, with an emphasis on adapting to the demands of the Unified Health System [23].

In this context, it is essential that teaching methodologies promote criticality, reflection, and humanistic skills and attitudes in students [22]. Teaching about the health of the black population becomes invaluable and challenging for universities and professors, from historical, social, pedagogical, and ethical perspectives.

Racism is widely spread in society, and that is not different in education. From basic to higher education, in public or private sectors, it pervades students and teachers’ relations, curricula, and institutional infra- and superstructure. It, thus, contributes to maintaining an elite-dominated and prejudicial social status quo [24].

The ethnic–racial issue must be widely debated, beyond curriculum components that discuss deprivation, as in the case presented in this paper. The academic universe is poor of popular universities that promote views of liberty and anti-racism as well as an ethnic/racial responsibility. It is urgent that knowledge production stop reproducing the coloniality of knowledge and start promoting the cultural and academic respect to and valuation of indigenous and black ancestry [24].

In health, students and professionals, with an antiracist education and attitude, contribute to break through access barriers, reduce maternal and neonatal mortality, reduce violence, and mitigate other conditions that lead to illness in that population. When well-trained physicians understand the needs and priorities of the black population, they act for the sake of attention, care and health rights [24].

The University of Brasilia School of Medicine reformulated the medical curriculum guided by competency domains: Knowledge, Skills, and Attitudes. Those domains cover themes of black population health, with the objective of discussing racism as a health condition; epidemiology of health problems in the black population; role of the family and community doctor in confronting inequities; and anti-racist practices in health [22].

## 5. The Experience of the University of Brasilia

The experience of the University of Brasilia School of Medicine has shown that students, when provoked to reflect on structural racism, manage to produce critical reviews of excellent quality, in which they articulate the content of classes with health system policies, ethical and existential issues of the doctor–patient relationship, plus aspects of clinical communication and subjective experiences.

From the professors’ point of view, the main focus in the training of health professionals is to deal with the racial issue in health. Since 2018, professors have observed that during the classes and in the final exam, the students show that they understand the influence of black race issues in the environmental, organic, and genetic domains. In addition, they incorporated the race–color category in clinical reasoning during the clinical interview. 

All professors participated in deeper discussions about what the curriculum entails, and they suggested that pedagogical investments should focus on the dialogue between medicine and social sciences. This can expand the students’ theoretical framework, generating new questions and exchanges among professors from medicine and social sciences, as well as expanding critical perspectives in medical training and in life in society.

The experience of training participants and the students’ feedback on teaching can be summarized in two points: (1) training was important to understand how to incorporate racial issues in health and (2) the pedagogical investment in different types of teaching materials (articles, policies, films, podcasts, books, and visual arts) was an important stimulus for the study of content.

The incorporation of the anti-racist lens in clinical reasoning represents the integral care of the patient and the confrontation of inequities, valuing their epidemiological context and their life narrative [6]. The clinical relationship must apprehend the subject and their world, allowing for the patient’s subjectivation in the act of care, and for the professional being able to name racism as a factor of illness, based on the concrete investigation of the patient’s suffering [12]. 

## 6. Conclusions

Changing the curriculum, infrastructure, and institutional superstructures for the incorporation of anti-racist practices in health education is a challenge. At the University of Brasilia, we want the ethnic–racial issue to permeate all curricular components of medical education.

Finally, pedagogical investments aimed at the dialogue between medicine and social sciences can broaden the theoretical framework of students, as well as critical perspectives in medical education and in life in society. An environment of profound exchanges in the teaching–learning process by adopting anti-racism praxis as a competency in the medical curriculum is a paradigm shift in favor of reducing and combating racial inequities.

## Data Availability

Not applicable.
